# *PTCH1* ^+/−^ Dermal Fibroblasts Isolated from Healthy Skin of Gorlin Syndrome Patients Exhibit Features of Carcinoma Associated Fibroblasts

**DOI:** 10.1371/journal.pone.0004818

**Published:** 2009-03-16

**Authors:** Alexandre Valin, Stéphanie Barnay-Verdier, Thomas Robert, Hugues Ripoche, Florence Brellier, Odile Chevallier-Lagente, Marie-Françoise Avril, Thierry Magnaldo

**Affiliations:** 1 CNRS FRE2939, Université Paris Sud-Institut Gustave Roussy, Villejuif, France; 2 INSERM UMRS872, Université Paris 5/6-Centre de recherche des Cordeliers, Paris, France; 3 CNRS Unité de génomique fonctionnelle, Université Paris Sud-Institut Gustave Roussy, Villejuif, France; 4 Biomedical Research, Friedrich Miescher Institute, Basel, Switzerland; 5 Service de Dermatologie, Université Paris 5-APHP, Paris, France; Tufts University, United States of America

## Abstract

Gorlin's or nevoid basal cell carcinoma syndrome (NBCCS) causes predisposition to basal cell carcinoma (BCC), the commonest cancer in adult human. Mutations in the tumor suppressor gene *PTCH1* are responsible for this autosomal dominant syndrome. In NBCCS patients, as in the general population, ultraviolet exposure is a major risk factor for BCC development. However these patients also develop BCCs in sun-protected areas of the skin, suggesting the existence of other mechanisms for BCC predisposition in NBCCS patients. As increasing evidence supports the idea that the stroma influences carcinoma development, we hypothesized that NBCCS fibroblasts could facilitate BCC occurence of the patients. WT (n = 3) and NBCCS fibroblasts bearing either nonsense (n = 3) or missense (n = 3) *PTCH1* mutations were cultured in dermal equivalents made of a collagen matrix and their transcriptomes were compared by whole genome microarray analyses. Strikingly, NBCCS fibroblasts over-expressed mRNAs encoding pro-tumoral factors such as Matrix Metalloproteinases 1 and 3 and tenascin C. They also over-expressed mRNA of pro-proliferative diffusible factors such as fibroblast growth factor 7 and the stromal cell-derived factor 1 alpha, known for its expression in carcinoma associated fibroblasts. These data indicate that the *PTCH1^+/−^* genotype of healthy NBCCS fibroblasts results in phenotypic traits highly reminiscent of those of BCC associated fibroblasts, a clue to the yet mysterious proneness to non photo-exposed BCCs in NBCCS patients.

## Introduction

Non melanocytic skin cancers are the most prevailing cancers in human and 80 percent of them are basal cell carcinomas (BCCs) [1], [2]. BCC is the commonest cancer in adult human; its incidence has been increasing constantly during the last 50 years in the general population [3], [4]. The Gorlin syndrome is an autosomal dominant genetic disease, also named nevoid basal cell carcinoma syndrome (NBCCS). NBCCS is associated to a dramatic predisposition to BCCs (up to hundreds) [5]. Other clinical features include various developmental traits and, in 3 to 5 percent patients, susceptibility to medulloblastoma. In 1996, mutations in the tumor suppressor gene *PATCHED* (*PTCH1*) were found to be associated to NBCCS [6], [7]. Most *PTCH1* germinal mutations lead to premature stop codon [8], and in BCCs, are accompanied by somatic mutations or loss of heterozygosity (LOH) at the *PTCH1* locus (9q22.3) [9], [10], as expected for a tumor suppressor gene [11]. In sporadic BCCs somatic mutations in *PTCH1* have been reported in up to 67% of cases; most of them correspond to ultraviolet fingerprints, C→T and CC→TT transitions [12]–[14]. Sporadic BCCs also display frequent (93% cases) LOH of the *PTCH1* locus [15], [16].

The PATCHED protein acts as the receptor of the diffusible morphogen SONIC HEDGEHOG (SHH). Binding of SHH to PATCHED relieves its inhibitory effect on the pathway activation, leading to the transcription of target genes including *PTCH1* itself and glioma-associated oncogene homolog transcription factors 1 and 2 (*GLI1/2*).

Bi-allelic inactivating mutations of *PTCH1* in both sporadic and NBCCS BCCs presumably lead to SHH-independent constitutive activation of the pathway as suggested by over-expression of the targets genes, *PTCH1* itself and *GLI1* [12], [17]–[20]. Thus, loss of control of the SHH pathway appears as a hallmark of BCC. Together, these observations underline the essential role of maintenance of the SHH pathway in human skin homeostasis.

In the general population BCCs develop almost exclusively in sun-exposed areas of the skin [2]. In contrast, and, intriguingly, NBCCS BCCs are observed in both sun-protected and sun-exposed areas. Interestingly, our previous studies have shown that both fibroblasts and keratinocytes from NBCCS patients exhibit normal nucleotide excision repair of UVB-induced DNA lesions and survival capacities following a single UVB irradiation [21]. These data suggest that solar UVs are far from being the only etiologic factor of BCC in NBCCS patients. Other mechanisms such as altered dermo-epidermal interactions could contribute to development of NBCCS BCC in non photo-exposed skin [22]. More and more evidence accumulates indicating the role of the stroma in tumoral development in general [23], and in the particular case of BCC as well.

Here, we hypothesized that NBCCS fibroblasts could exhibit features determinant towards BCC development. We compared the genome expression of NBCCS primary fibroblasts cultured in a dermal equivalent to that of control fibroblasts under the same circumstances. Results indicate that essential factors for BCC development, such as MMP1, MMP3, CXCL12, ANGPTL2, MGP, TNC, and SFRP2 are over-expressed by NBCCS fibroblasts. Hence, heterozygous *PTCH1* mutations may result in expression of genes whose stromal expression is associated with (skin) cancer development. Our data strongly suggest that NBCCS fibroblasts could play a prominent role in predisposition of patients towards BCCs, including in sun-protected skin areas.

## Results

### Whole genome microarray comparison of control and NBCCS fibroblasts transcriptomes

We compared the genome wide expression profile of 9 primary independent fibroblasts strains cultured in dermal equivalents (CTRL, n = 3; NBCCS, n = 6). Three NBCCS fibroblast strains bore missense mutations and 3 bore nonsense mutations in the *PTCH1* gene. Total RNAs were extracted from the dermal equivalents and gathered in 3 pools according to the genetic status of *PTCH1* (WT; missense and nonsense *PTCH1* mutations). The transcription profile of the control pool was compared to that of each NBCCS pool, on Agilent® human whole genome oligo microarray. Results were analyzed with the Rosetta Resolver**®** system for gene expression analysis [24].

**Figure 1:**
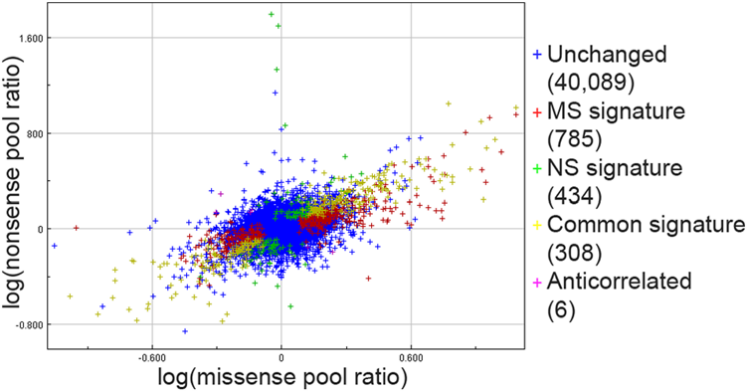
Comparison plot of the missense and the nonsense signatures revealed by the microarray analysis. All the genes with statistically (p<10^−5^) different mRNA level between any NBCCS pool and the control pool are plotted. In abscissa: the logarithm of the ratio of the intensities in the missense and the control pools. In ordinate: the logarithm of the ratio of the intensities in the nonsense and the control pools. Note the correlation between the missense (MS) signature and the nonsense (NS) signature revealed by the linear shape of the common signature (correlation coefficient of 0.929).

Results of this genomic screen are available in the database ArrayExpress (accession number: E-TABM-549). To select the genes with a high probability to be differentially expressed in the NBCCS pools, the threshold for the p-value was set to 10^−5^. 182 genes were found up-regulated and 126 down-regulated (p-value<10^−5^) in both the missense and the nonsense pools (Table S1). These 308 genes of the common signature displayed a high correlation between the two NBCCS pools (correlation coefficient of 0.929) (Figure 1). Only 6 probes corresponding to 5 genes were anti-correlated, i.e. up-regulated in one NBCCS pool and down-regulated in the other one (*CILP*, *LY6K*, *CLEC3B*, *CYP1B1* and *LEPROT*; Table S2). For all the primary sequences, an analysis of variance (ANOVA) of the logarithm of the ratio of the intensities in each NBCCS pool to the WT pool was performed. Cluster analysis was done on the ANOVA results. 38 genes, including the 5 anti-correlated genes, with different levels of expression in the two NBCCS pools are listed and clustered in Figure S1 and Table S3. These data indicate for the first time that missense or nonsense mutations of *PTCH1* have overall very similar consequences on the transcriptome of dermal fibroblasts.

Then, we focused more precisely on the genes whose mRNAs amounts were increased by at least 2.1 in both the missense and the nonsense pools (Table 1).

**Table 1:** More than 2.1 fold up-regulated genes in the two NBCCS pools*.

Primary Sequence Name	Accession Number	missense pool		nonsense pool		NBCCS	
		Fold Increase	p-value	Fold Increase	p-value	Fold Increase	p-value
MMP3	NM_002422	19.7421	0	13.0139	0	15.931	0
ALDH1A3	NM_000693	5.92	0	10.883	0	6.799	1.61E-34
KCNJ8	NM_004982	12.2448	0	10.1035	5.14E-32	11.282	0
MMP1	NM_002421	8.4122	0	7.8028	0	8.174	0
CLIC6	NM_053277	18.5676	1.72E-35	7.711	3.54E-13	17.042	3.64E-31
COL11A1	NM_080629	9.7967	9.06E-22	5.4611	4.28E-14	10.146	2.21E-12
IL13RA2	NM_000640	8.9084	0	4.6296	2.00E-19	6.822	1.52E-23
DHRS3	NM_004753	4.3342	0	3.7553	3.85E-33	4.409	0
APBB1IP	NM_019043	4.6804	0	3.7532	2.47E-40	4.209	0
MASP1	NM_139125	3.2252	1.34E-21	3.421	3.33E-18	3.245	4.03E-21
BX537788	BX537788	4.3842	9.63E-37	3.408	1.22E-15	4.197	4.80E-19
SPOCK1	NM_004598	3.1251	0	3.3341	0	3.376	0
ANGPTL4	NM_139314	7.5098	0	3.1122	0	4.353	1.07E-10
GHR	NM_000163	4.5814	9.60E-26	3.0934	4.03E-06	4.065	5.44E-16
KCTD15	NM_024076	3.0865	1.44E-21	3.0498	1.42E-19	3.069	0
AI476245	AI476245	5.1206	0	3.0481	3.41E-17	4.46	4.73E-33
AF264625	AF264625	2.8355	7.77E-28	2.8518	2.37E-25	2.918	0
SMOC2	NM_022138	5.5702	6.90E-20	2.788	4.85E-06	5.133	2.20E-13
FLJ14834	NM_032849	5.8414	0	2.7641	6.60E-32	3.872	1.26E-11
MGC42157	BC030111	2.9857	0	2.759	1.59E-22	2.986	0
PTGIS	NM_000961	2.8252	5.37E-22	2.7335	2.82E-23	2.882	1.10E-42
NANOS1	NM_199461	3.609	3.75E-13	2.6956	5.74E-12	2.696	5.75E-12
DPP3	NM_130443	2.6339	8.64E-18	2.6929	3.39E-17	2.663	5.37E-38
CXCL12	NM_199168	2.8062	1.10E-34	2.5629	5.33E-25	2.695	7.25E-38
MGST1	NM_145791	3.9293	0	2.5332	2.55E-24	2.903	1.35E-13
SNCA	NM_007308	3.0297	3.71E-30	2.5018	7.41E-09	2.853	1.17E-26
PRL	NM_000948	5.4189	0	2.484	2.42E-15	4.168	1.90E-11
ADARB1	NM_015833	2.2018	6.96E-29	2.4494	6.38E-38	2.333	0
AKR1C1	NM_001353	2.8523	5.59E-37	2.4218	7.18E-21	2.53	1.65E-29
CCRL1	NM_178445	2.3757	1.12E-18	2.4162	1.81E-26	2.484	0
BC035260	BC035260	2.1041	3.96E-12	2.4093	6.50E-09	2.189	2.68E-21
AA417913	AA417913	2.37	3.52E-19	2.409	1.49E-29	2.429	5.32E-38
TFPI2	AK129833	6.1688	4.05E-40	2.4071	1.13E-06	5.176	3.85E-11
GPC6	BX640888	3.4137	7.47E-36	2.405	7.60E-21	2.627	2.08E-22
DSCR1	NM_004414	2.2516	1.42E-10	2.4037	1.24E-11	2.322	3.13E-22
STEAP1	NM_012449	2.9091	0	2.3808	7.46E-35	2.545	8.36E-35
AKR1C3	NM_003739	2.3671	8.04E-21	2.3569	7.55E-24	2.362	0
AKR1C1	NM_001353	2.8883	0	2.3474	1.23E-39	2.682	4.93E-13
CMTM1	NM_181289	2.3129	3.71E-31	2.3448	1.11E-25	2.368	0
EXOSC6	NM_058219	2.2792	0	2.3187	1.12E-44	2.275	0
HSPA5BP1	NM_017870	2.8592	5.23E-18	2.2717	4.12E-15	2.354	1.88E-30
CDON	AK022986	2.6989	2.02E-19	2.2717	1.60E-08	2.452	5.46E-14
STEAP2	NM_152999	2.8101	7.94E-37	2.2325	1.10E-09	2.714	0
PPAP2A	NM_176895	3.5108	6.87E-38	2.23	1.83E-35	2.49	3.14E-16
STEAP1	NM_012449	2.8538	0	2.2024	2.10E-39	2.392	3.50E-23
CPM	NM_001874	3.715	0	2.1577	1.14E-25	3.001	3.31E-11
NR4A3	NM_173200	4.2339	0	2.1442	3.44E-11	3.204	2.21E-10
CA12	NM_001218	2.9836	0	2.1333	0	2.344	6.78E-19
PCSK5	NM_006200	3.4846	0	2.1278	1.36E-09	2.602	2.16E-08
WISP2	NM_003881	2.5103	1.30E-23	2.1266	6.04E-18	2.185	2.91E-35
SAA1	NM_000331	3.048	0	2.1112	1.25E-21	2.343	4.35E-16

* List of the 51 genes, with their OMIM accession number associated, whose mRNA levels were increased more than 2.1 fold in the missense and the nonsense pools. In the two NBCCS pools, for each gene, the fold increase compared to the control pool and its p-value are mentioned. The average NBCCS level was determined by combining the two NBCCS pools in the Resolver software. The p-value “0” is due to rounding of p-values strictly inferior to 7.01*10^−45^.

### Over-expression of pro-tumoral matrix metalloproteinases and modified expression of extracellular matrix and basement membrane components in NBCCS fibroblasts

By applying SBIME (Searching for a Biological Interpretation of Microarray Experiments) [25] to the data with the Gene Ontology annotations, we found that genes involved in the extracellular matrix composition or remodeling, and genes involved in cell adhesion were overrepresented in the signature of both NBCCS pools. mRNA of the matrix metalloproteinase 3 (*MMP3*; stromelysin 1) exhibited the highest increased level in both NBCCS pools. *MMP3* mRNA increased by 19.7 and 13 in the missense and the nonsense pools, respectively, compared to the control pool (Table 1). mRNA of the matrix metalloproteinase 1 (*MMP1*; collagenase 1) increased by 8.4 and 7.8 in the missense and the nonsense pools, respectively (Table 1).

**Figure 2:**
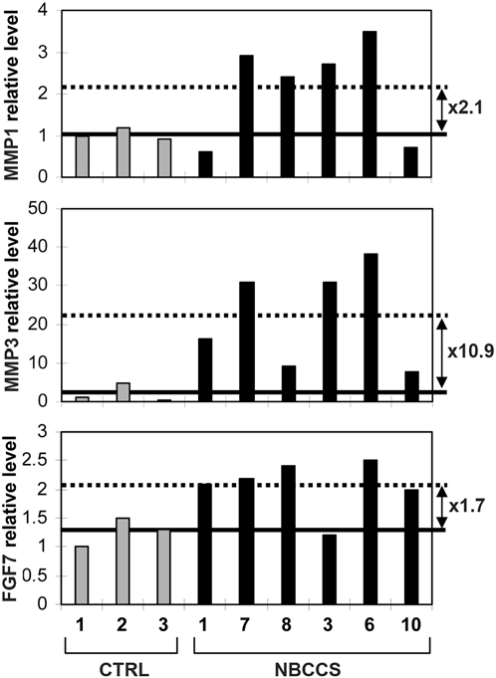
NBCCS fibroblasts in dermal equivalents over-express MMP1, MMP3 and FGF7. The levels of secreted MMP1, MMP3 and FGF7 were determined by ELISA in the supernatants of the dermal equivalents used for the microarray assay. Results were set to 1 for the protein levels in the supernatant of the dermal equivalent with control (CTRL) fibroblast strain 1. The protein level in control strains is represented with grey histogram and with black histogram in NBCCS strains. The average MMPs and FGF7 levels in the supernatants of control (NBCCS) dermal equivalents are indicated with a plain (dotted) line. The ratios between the average protein levels in NBCCS and in control fibroblasts are indicated (arrow).

To confirm microarray results, reverse transcription and quantitative polymerase chain reactions (RT-QPCR) were performed on each mRNA sample extracted from the 3 control and 6 NBCCS fibroblast primary strains used in the genomic screen. The average *MMP3* and *MMP1* mRNA levels were found statistically increased by 26.84 and 8.43, respectively, in NBCCS compared to control fibroblasts (p<0.025 for both *MMP3* and *MMP1*) (Table 2). We also noticed by ELISA increased levels of MMP1 and MMP3 secreted proteins in culture supernatants of NBCCS dermal equivalents by 10.9 and 2.1 respectively (Figure 2). These findings indicate that *MMP3* and *MMP1* mRNA increases result in over-production and secretion of their respective protein.

**Table 2:** RT-QPCR validation of microarray results/Relative level of up and down-regulated mRNA in control and NBCCS fibroblasts*.

Gene Name	CTRL pool			missense pool			nonsense pool			average NBCCS/average CTRL	NBCCS <or> CTRL p-value
	CTRL1	CTRL2	CTRL3	NBCCS1	NBCCS7	NBCCS8	NBCCS3	NBCCS6	NBCCS10		
MMP1	1	1.3	3.62	10.39	30.98	5.17	24.82	23.65	4.9	8.43	p<0.025
MMP3	1	0.89	2.32	40.98	77.08	30.01	16.18	45.77	16.45	26.84	p<0.025
COL3A1	1	0.64	0.67	0.35	0.53	0.6	0.25	0.54	0.81	0.66	p≤0.05
COL7A1	1	1.67	1.45	0.45	0.57	0.74	1.38	0.48	1.19	0.58	p≤0.05
COL11A1	1	0.11	1.99	15.93	5.62	22.91	0.02	10.93	15.73	11.47	NS
LAMA2	1	0.57	0.54	0.34	0.25	0.34	0.21	0.32	0.34	0.43	p<0.025
TNC	1	1.03	0.99	1.46	1.29	1.27	0.61	0.77	2	1.22	NS
CXCL12	1	1.13	1.22	2.46	2.77	2.48	1.77	4.24	2.31	2.39	p<0.025
MGP	1	0.01	0.18	4.67	1.38	2.65	0.27	1.28	2.11	5.19	p<0.025
ANGPTL2	1	0.98	0.93	3.32	2.09	3.21	1.16	3.78	3.67	2.95	p<0.025
ANGPTL4	1	0.8	0.79	12.24	7.2	15.1	2.08	10.18	3.75	9.77	p<0.025
FGF7	1	1.76	2.07	4.56	2.63	4.48	1.32	5.09	3.75	2.26	p≤0.05
GREM1	1	2.03	4.54	3.86	1.57	8.36	1.92	5.73	4.25	1.7	NS
SFRP2	1	0.65	0.49	1.66	0.73	4.04	1.6	2.71	4.08	3.46	p<0.025
DKK3	1	0.58	1.06	0.1	0.21	0.13	0.38	0.19	0.36	0.26	p<0.025
WNT5A	1	0.98	1.89	0.52	0.39	1.05	0.42	0.74	0.69	0.5	p≤0.05
WISP2	1	0.35	0.32	0.71	2.08	1.73	0.77	1.94	1.93	2.74	p≤0.05
ID2	1	1.26	2.92	3.39	3.65	7.26	1.38	8.27	3.2	2.62	p<0.025

* The level of mRNA of the indicated genes was measured by RT-QPCR on the mRNAs extracted from each dermal equivalent used for the microarray assay (CTRL: n = 3; NBCCS: n = 6). Results were normalized and set to 1 for the mRNA from the dermal equivalent with control fibroblast strain 1. The ratios between the average mRNA levels in the NBCCS and the control fibroblasts and the p-value are indicated. NS: not significant (p>0.05).

**Figure 3:**
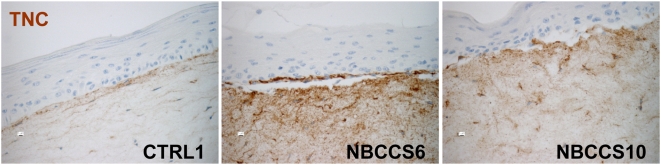
NBCCS fibroblasts in organotypic cultures over-express TNC. Organotypic skin cultures with control keratinocytes and the indicated fibroblasts were developed and 5 µm paraffin sections were immunolabelled using anti-human TNC antibody. Note the barely detectable labelling of TNC in control dermis and its increase in both NBCCS fibroblast strains tested (6 and 10).

The mRNAs of some of the components of the extracellular matrix (ECM) were also found up-regulated in the missense and the nonsense NBCCS pools. For instance, the amount of collagen type 11 alpha 1 (*COL11A1*) mRNA was increased by 9.8 and 5.5 and tenascin C (*TNC*) mRNA was increased by 1.4 and 1.3 in the missense and the nonsense pools, respectively (Table 1 and Table S1). RT-QPCR confirmed the increased level of *COL11A1* mRNA in 5 of the 6 NBCCS fibroblasts compared to control fibroblasts. The average rate of *COL11A1* mRNA over-expression in NBCCS fibroblasts was 11.47 (Table 2). The slight increased level of TNC mRNA was confirmed by RT-QPCR (1.22 fold; Table 2) and immunohistochemistry performed on organotypic skin cultures comprising fibroblasts isolated from two independent NBCSS patients studied here revealed a stronger TNC over-expression (Figure 3).

The mRNA levels of some components of the basement membrane were decreased in the missense and the nonsense NBCCS pools. Collagen type 3 alpha 1 (*COL3A1*) were decreased by 1.4 and 1.5 in the missense and the nonsense pools, respectively (Table S1). Similarly the collagen type 7 alpha 1 (*COL7A1*) mRNA level was decreased by 1.8 and 1.3 in the two NBCCS pools (Table S1 and in ArrayExpress). The mRNA level of the laminin alpha 2 (*LAMA2*) was also decreased by 1.5 and 1.4 respectively in the missense and the nonsense pool (Table S1). RT-QPCR confirmed the decreased average mRNA amounts of *COL3A1*, *COL7A1*, and *LAMA2* by 1.52, 1.72 and 2.33 respectively (p≤0.05; p≤0.05; p<0.025), in NBCCS compared to control fibroblasts (Table 2).

Together these results indicate that some ECM and basement membrane components are expressed differentially between NBCCS and control fibroblasts under organotypic culture conditions.

### NBCCS fibroblasts over-express the BMP antagonist GREMLIN1, growth factor and cytokines associated with BCC stroma

The mRNA levels of chemokine (C-X-C motif) ligand 12 (*CXCL12*; stromal-cell derived factor 1 alpha) and of the bone morphogenetic protein (BMP) antagonist GREMLIN1 (*GREM1*) were found up-regulated in our microarray analysis, as well as those of angiopoietin like 2 and 4 (*ANGPTL2*; *ANGPTL4*), matrix GLA protein (*MGP*) and fibroblast growth factor 7 (*FGF7*; keratinocyte growth factor). The mRNA level increases, in the missense and the nonsense pools, were respectively 2.8 and 2.6 for *CXCL12*, 7.5 and 3.1 for *ANGPTL4* (Table 1). Similarly, the increases of *MGP* mRNA levels were 2 and 1.4 in the missense and the nonsense pools respectively, 2 and 1.7 for *ANGPTL2*, 1.9 and 1.7 for *GREM1* and finally 2.3 and 1.6 for *FGF7* (Table S1).

RT-QPCR confirmed that *CXCL12*, *MGP*, *ANGPTL2*, *ANGPTL4* and *FGF7* average mRNA levels were increased in NBCCS fibroblasts by 2.39, 5.19, 2.95, 9.77 and 2.26 respectively (p<0.025 for each; Table 2). *ANGPTL4* mRNA level was higher in fibroblasts of the missense pool than in fibroblasts of the nonsense pool. The increase of *GREM1* mRNA level in NBCCS fibroblasts did not reach statistical significance.

**Figure 4:**
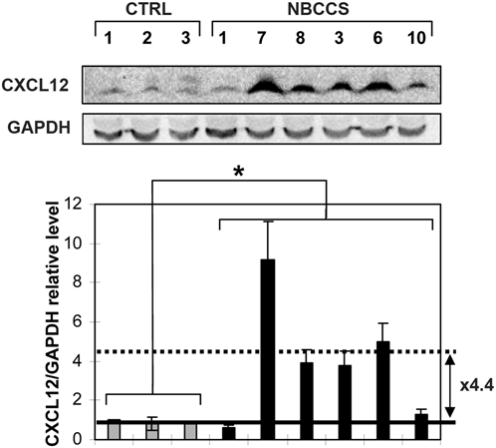
NBCCS fibroblasts over-express CXCL12. Western blot analysis of CXCL12 was performed on cellular extracts of the 3 control (CTRL) and 6 NBCCS fibroblast strains. GAPDH was used as a loading control. The level of CXCL12 was normalized to the GAPDH level and was set to 1 in the control fibroblasts strain 1. A representative western blot and quantification from 3 independent experiments are represented. Error bars refer to standard errors. The protein level in control strains is represented with grey histogram and with black histogram in NBCCS strains. The average CXCL12 level in control (NBCCS) fibroblasts is indicated with a plain (dotted) line (*: p≤0.05). The ratio between the average mRNA level in NBCCS and in control fibroblasts is indicated (arrow).

These mRNA over-expressions were correlated with increased levels of protein as confirmed by western blot analysis on cellular extracts for CXCL12 (4.4 average fold increase; p≤0.05) (Figure 4) and by ELISA on the supernatant of dermal equivalents for FGF7 (1.7 fold increase) (Figure 2).

### The WNT/beta catenin pathway in NBCCS fibroblasts

The WNT/beta catenin pathway plays a crucial role in the regulation of proliferation and differentiation of keratinocytes [26]. In BCCs, nuclear beta catenin staining, corresponding to pathway activation, has been observed and associated with increased proliferation [27], [28]. Among the secreted inhibitors of the WNT/beta catenin pathway, Dickkopf 1 (*DKK1*), secreted frizzed related protein 1 (*SFRP1*), *SFRP2*, and WNT inhibitory factor 1 (*WIF1*) were up-regulated while *DKK3* was down-regulated (Table S1 and in ArrayExpress). RT-QPCR confirmed the increased level of *SFRP2* mRNA (by 3.46; p<0.025) and the decreased level of *DKK3* mRNA (by 3.85; p<0.05) in NBCCS fibroblasts (Table 2). *WNT5A* mRNA, which encodes a ligand of the non canonical WNT pathway, was down-regulated by 2.9 and 2.7 in the missense and the nonsense pool respectively (Table S1). A significant decreased level in NBCCS fibroblasts was confirmed by RT-QPCR (average 2.0 fold decrease; p≤0.05; Table 2).

The mRNA levels of two target genes of WNT, WNT1 Inducible Signaling Pathway Protein 2 (*WISP2*) and Inhibitor of DNA Binding 2 (*ID2*) were increased in both NBCCS pools (Table 1 and Table S1). RT-QPCR confirmed that *WISP2* and *ID2* mRNA levels were significantly increased by 2.74 (p≤0.05) and 2.62 (p<0.025) respectively in NBCCS fibroblasts (Table 2).

## Discussion

The study of carcinoma development has long been mainly focused on tumor suppressor genes mutations and proto-oncogenes activations in tumor cells. However, increasing evidence has indicated that tumor-stroma interactions regulate tumoral growth and invasiveness, and contribute to metastasis as reviewed in [29]–[31]. In the case of BCCs, the stroma seems to be essential for the survival of cancer cells. Indeed, by contrast to squamous cell carcinoma cells, BCC cells can hardly be cultured *ex vivo*, and exhibit virtually null metastatic potential *in vivo* [32]. Grafting experiments have shown that growth of BCC cells depends on the presence of their carcinoma associated fibroblasts (CAF) [33], [34]. In the present study, using whole genome transcriptome analyses, we have investigated the extent to which *PTCH1^+/−^* dermal fibroblasts could contribute to BCC development providing that NBCCS tumors also develop in non photo-exposed skin areas, i.e. in the absence of external genotoxic stress. Although isolated from healthy skin of patients, NBCCS fibroblasts exhibit features very close to those of BCC-associated fibroblasts from non NBCCS individuals.

Perhaps, the most intriguing observation of our genomic screen was that none of the members of the SHH pathway was differentially expressed in NBCCS compared to control fibroblasts. In vertebrates, skin homeostasis relies on a balanced dialog between dermal and epidermal cells [35]–[37]. Experimental models suggest that the SHH pathway is involved not only in the development and cycle of hair follicles [38] but also in the regulation of recruitment and proliferation of interfollicular epidermal stem cells [39], [40]. Loss of control and activation of the SHH pathway was found in all BCCs tested [12], [17]–[20]. Thus, beyond its evident role in the control of growth of epidermal cells, proper control of the SHH pathway could also contribute to dermo-epidermal interactions, a hypothesis fitting well with distal transmission of the SHH signal between expressing and receiving cells [41] and with growth features of BCC cells as well.

As for members of the SHH pathway, our results did not contribute to a clear conclusion on either activation or inhibition of the WNT pathway in NBCCS fibroblasts. Increased mRNAs levels of *SFRP2* and *WNT5A*, have been found in CAF of BCC [42] and in whole BCC [43] respectively. *SFRP2* mRNA was also found increased in our screen. In contrast, mRNA amounts of *WNT5A* and of *DKK3* which encodes another inhibitor of the WNT pathway were decreased (Table 2). Some target genes of the WNT pathway, *WISP2* and *ID2* were up-regulated (Table 2) while others such as C-*MYC* and *CYCLIN D1* were not. *WISP2* has been shown to be strongly expressed in the stroma of breast tumors in Wnt1-transgenic mice [44]. ID2 was over-expressed in human colorectal carcinomas [45].

The absence of epidermal cells in our experimental settings may perhaps explain why, first, target genes of the SHH pathway were not found differentially expressed in NBCCS compared to control fibroblasts and second, the non-conclusive pictures drawn from study of the WNT pathway. Rather, our screening strategy may reflect cell autonomous impact of heterozygous *PTCH1* mutations in mesenchymal cells. Future experiments in the presence of both fibroblasts and keratinocytes should shed light on these issues.

For the first time, comparison of the whole genome expression in NBCCS and control fibroblasts clearly reveals a signature including several genes known for their association with tumor growth and invasiveness. A previous study reported over-expression of *MMP3* but not of *MMP1* mRNAs in cultured NBCCS fibroblasts and NBCCS BCCs [46]. Here, increases in MMP3 and MMP1 mRNAs and proteins amounts, were reported in NBCCS fibroblasts cultured in dermal equivalents (Table 2 and Figure 2). Interestingly over-expression of these MMPs has been reported in CAF of sporadic BCCs [47], [48]. MMP1 over-expression was also observed in dermal fibroblasts from xeroderma pigmentosum group C patients and is thought to be involved in skin cancer development in these patients [49]. Epidermal over-expression of MMP1 in transgenic mice induces epidermal hyperplasia [50]. Also, MMP3 over-expression in mouse mammary epithelial cell line stimulates the epithelial to mesenchyme transition and invasiveness [51]. Most interestingly, a recent report from Dr. Bissell's laboratory indicated that MMP3-induced epithelial to mesenchyme transition and genomic instability are mediated through increased production of reactive oxygen species [52]. Since, first, MMP1 processing is in part dependent on MMP3, second, these MMPs present partially distinct substrate specificities, and, third, none of their tissue inhibitors was found over-expressed, concomitant increases of MMP1 and MMP3 in patients' fibroblasts strongly argue for a prominent role of the mesenchyme in BCC development and invasiveness in NBCCS.

NBCCS fibroblasts also over-expressed the pro-tumoral ECM component TNC (by 1.22, Table 2; Figure 3). TNC was found strongly expressed in the stroma of BCCs [53] and can up-regulate *MMP3* expression [54]. Interestingly, TNC is a proteolysis substrate of MMP1 and MMP3 [55]. In addition, TNC contains EGF-like repeats whose binding to the EGF receptor stimulates mitogenesis [56]. Our study also shows that mRNA amounts of *CXCL12*, *GREM1* and *FGF7* (Table 2, Figure 2 and Figure 4) were also significantly increased in NBCCS fibroblasts. CXCL12 is expressed by CAF of BCC, is involved in the invasion of BCC cells [57] and can also recruit endothelial cells [58], [59]. GREM1 over-expression is associated with BCC stromal cells and promotes BCC cells proliferation [60]. FGF7 (keratinocyte growth factor) has a mitogenic activity in keratinocytes [61]. In NBCCS patients, presence of high levels of MMP1 and MMP3, as well as growth regulatory factors such as TNC, CXCL12, GREM1 and FGF7 could stimulate proliferation of BCC cells and elicit invasiveness. COL11A1 mRNA was also found increased in NBCCS fibroblasts, an observation reminiscent of its over-expression by stromal fibroblasts in human colorectal tumors [62]. In contrast lower amounts of mRNAs of the basement membrane components *COL3A1*, *COL7A1* and *LAMA2* [63] (Table 2) could be responsible for loosening basement membrane and, hence, facilitate local aggressiveness of pre-tumoral (*PTCH1^+/−^* keratinocytes) or tumoral epidermal cells [22].

Finally, the 3 times increased mRNA level of *ANGPTL2* which encodes a member of the angiopoietin-like family (Table 2), could reflect pro-angiogenic properties as suggested by its effects on endothelial cells sprouting [64]. However, it was noteworthy that one of the most over-expressed mRNA by NBCCS fibroblasts in this study was *ANGPTL4* (9.7 fold increase; Table 1 and Table 2). ANGPTL4 can inhibit tumor cells (B16F0) motility and invasiveness [65] and exhibit anti-angiogenic properties [66]. Whether less vascularization [67] and very low metastatic potential of BCC compared to squamous cell carcinomas could be related to high levels of ANGPTL4 requires further investigations. Our data are in excellent agreement with those recently obtained by transcriptome analyses of CAF from BCC compared to perifollicular dermal fibroblasts [42]. In this study, the authors found that *SFRP2*, *ANGPTL2*, and *CXCL12* mRNA amounts were increased in CAF of BCC.

In summary, even if the causal link between *PTCH1^+/−^* genetic status and abnormal expression of genes reported here remains to be clarified, our data clearly converge towards a global trend of NBCCS dermal fibroblasts to express some characteristics of CAF from BCCs. Modified expressions of ECM and basement membrane components, which are also observed in the stroma of BCCs [68], suggest that dermal fibroblasts might also produce a BCC facilitating microenvironment in NBCCS patients. Importantly, all primary fibroblasts used in this study were isolated from biopsies of healthy and photo-protected skin and gave very similar results irrespective of the type of *PTCH1* mutation. Altogether, these results strongly suggest the involvement of dermal fibroblasts in BCC predisposition in NBCCS patients including in non photo-exposed skin areas. In addition, NBCCS keratinocytes from the same NBCCS patients exhibit spontaneous invasive properties in dermal equivalents populated with control fibroblasts [22]. More investigations must now be carried out in the long term to determine the extent to which NBCCS fibroblasts may affect the fate of epidermal keratinocytes. Also it would be essential to verify the present results in healthy, non photo exposed skin of NBCCS patient. Future studies should lead to improved approaches, taking into account the role of fibroblasts, in BCC prevention and/or treatment, not only in NBCCS patients but also in the general population.

## Materials and Methods

### Ethics Statement - Patients and cells

This study was approved by a local French ethic committee (CCPPRB: CSET935) and was conducted after informed written consent of NBCCS patients. Skin biopsies from NBCCS and control persons were taken from sun-protected healthy areas. NBCCS patients 1, 7 and 8 harbour independent missense mutations in *PTCH1*; NBCCS patients 3, 6 and 10 harbour nonsense independent mutations in *PTCH1*. NBCCS patients are described in [21] and in ArrayExpress. From these patients, primary dermal fibroblasts were cultured as described before [69]. Experiments were performed using cells at passages 5 to 9.

### Dermal equivalents and organotypic skin cultures

All fibroblasts (WT n = 3; NBCCS n = 6) were cultured in a tridimensional type I collagen matrix called dermal equivalent. Dermal equivalents and organotypic skin cultures were cultured in immersion and then at the air-liquid interface as described in [70]. For dermal equivalents keratinocytes were replaced by culture medium. Organotypic skin cultures were realized using control keratinocytes at passage 5 with CTRL1, NBCCS6 or NBCCS10 fibroblasts strains. These NBCCS strains, bearing independent nonsense mutations in *PTCH1*, were chosen as they are representative of the major type of mutation occurring in NBCCS patients.

### RNA extraction

Dermal equivalents were snap frozen in liquid nitrogen, ground to powder and then solubilized in TRIzol reagent (Invitrogen, Carlsbad, USA, CA). Then chloroform was added and the aqueous phase was removed. Total RNAs were precipitated in isopropanol and washed twice before being resuspended in nuclease free water. Total RNAs were purified using the RNA cleanup and concentration kit (QIAGEN, Hilden, Germany) and gathered in 3 pools according to the genetic status of *PTCH1* (WT; missense and nonsense *PTCH* mutations).

### Whole genome microarray analysis

Protocol is detailed in ArrayExpress (accession number: E-TABM-549). The mRNA pools were labelled using fluorescent low input linear amplification kit (Agilent, Santa Clara, USA, CA). Briefly, reverse transcription was performed using MMLV reverse transcriptase. Then, cyanine 3 or 5 labeled cRNAs were generated using T7 RNA polymerase. Hybridizations were carried out for 17 hours at 60°C with 1 µg of purified control and NBCCS probes on Agilent**®** human whole genome oligo microarray 44k. Slides were scanned using an Agilent 2565 AB DNAmicroarray scanner. Microarray images were analysed by using Feature extraction software version A.8.5.1.1. (Agilent). Raw data files were then imported into Resolver® system for gene expression data analysis (Rosetta Inpharmatics LLC, Seattle, USA, WA).

### RT-QPCR

Reverse transcription and quantitative real time PCR were performed as described in [22], using the specific primers detailed in supplementary Table S4. B2M, TBP, GAPDH, RPLO1 and PPIA were used as housekeeping genes. Results of the Q-PCR were normalized using the geNorm software (http://medgen.ugent.be/~jvdesomp/genorm/). A Mann-Whitney test was used to determine whether the levels of mRNA in the 3 control and 6 NBCCS dermal equivalents were statistically different.

### ELISA

MMP1 and MMP3 levels in the supernatant of the dermal equivalents were measured using the human biotrak assays (RPN2610 and RPN 2613, GE Healthcare, London, UK). FGF7 level was determined using the Human KGF Immunoassay (DKG00, R&D Systems, Minneapolis, Minnesota), according to the manufacturers' protocols.

### Immunohistochemistry

Tenascin C immunostainings were done on 5 µm paraffin sections of skin organotypic cultures with control keratinocytes and either CTRL1, NBCCS6 or NBCCS10, using the Discovery XT automated stainer (Ventana Medical Systems), with 3,3′-diaminobenzidine Map detection kit (Ventana). Antigen retrieval was achieved with a 4 minute incubation of Protease 2 (Ventana). Afterward, slides were incubated for 1 h at 37°C with mouse monoclonal anti-tenascin C (B28-13; 1∶50). Slides were then treated for 32 min at 37°C with a biotinylated universal secondary antibody (Ventana) and counterstained with hematoxylin and bluing reagent (Ventana).

### Western blot

Proteins (70 µg) from 2D fibroblast cultures were separated using a 12% SDS-PAGE, transferred onto polyvinyl difluoride membrane and probed with the rabbit polyclonal anti-SDF1 alpha (alias CXCL12) antibody (ab9797, Abcam, Cambridge, UK, 1/200). Membranes were then reprobed using the mouse monoclonal anti-GAPDH antibody (ab9484, Abcam,Cambridge, UK, 1/5000). Blots were revealed using electrochemiluminescence (ECL+) reagents (GE Healthcare, London, UK). The amount of CXCL12 was quantified, relative to that of GAPDH in a genegnome device, using the genetools software (Ozyme, Montigny-le-Bretonneux, France).

## Supporting Information

Figure S1 Cluster analysis of differentially expressed genes between the missense and the nonsense pools. Shown is the 2D cluster analysis of the ANOVA results of the microarray data (with p<10^−10^ as threshold). The log(ratio) for each slides of the dye-swap for the missense and nonsense pools are illustrated in red when the mRNAs are over-represented in the NBCCS pool compare to the control pool and conversely in green. The slides “missense and nonsense pools” marked with an asterisk (*) were incubated with Cy5 for the control target and Cy3 for the NBCCS target, and reciprocally for the slides without asterisk. Details on the genes and the ratios of intensity between NBCCS and control pools are included in Table S3.
(0.01 MB PDF)

Table S1 Common NBCCS signature of the microarray results: 308 genes differentially expressed (p<10^−5^) in the two NBCCS pools compared to the control pool. The microarray assay was performed in dye-swap. The results of the dye-swaps were combined for the missense and the nonsense pools. For each gene or sequence, the fold change and its associated p-value are mentioned. Positive fold changes stand for an increased expression in NBCCS pool; negative fold changes stand for a decreased expression in NBCCS pool.
(0.02 MB PDF)

Table S2 Anti-correlated genes between the missense and the nonsense pools. List of the genes up-regulated in one NBCCS pool and down regulated in the other among the genes with differential expression in NBCCS pools compare to the control pool (p<10^−5^). For each gene, the fold change and its associated p-value are mentioned. Positive fold changes stand for an increased expression in NBCCS pool; negative fold changes stand for a decreased expression in NBCCS pool.
(0.01 MB PDF)

Table S3 38 differentially expressed genes between the missense and the nonsense pools found by analysis of variance of the microarray results. For each slide of the dye-swaps, the fold change between NBCCS and control pools are indicated for the 38 genes differentially expressed between the two NBCCS pools. Positive fold changes stand for an increased expression in NBCCS pools; negative fold changes stand for a decreased expression in NBCCS pools. The slides “missense and nonsense pools” marked with an asterisk (*) were incubated with Cy5 for the control target and Cy3 for the NBCCS target, and reciprocally for the slides without asterisk.
(0.01 MB PDF)

Table S4 Primers used for quantitative real-time PCR. List of the TaqMan® Gene Expression Assays primers used for Q-PCR (Applied Biosystems, Foster City, USA, CA).
(0.01 MB PDF)
