# Supplementary figures and images for: PTCH1 +/− Dermal Fibroblasts Isolated from Healthy Skin of Gorlin Syndrome Patients Exhibit Features of Carcinoma Associated Fibroblasts
Source: PLoS One. 2009 Mar 16;4(3):e4818. doi: 10.1371/journal.pone.0004818 (PMC2654107; doi:10.1371/journal.pone.0004818)

Figure S1

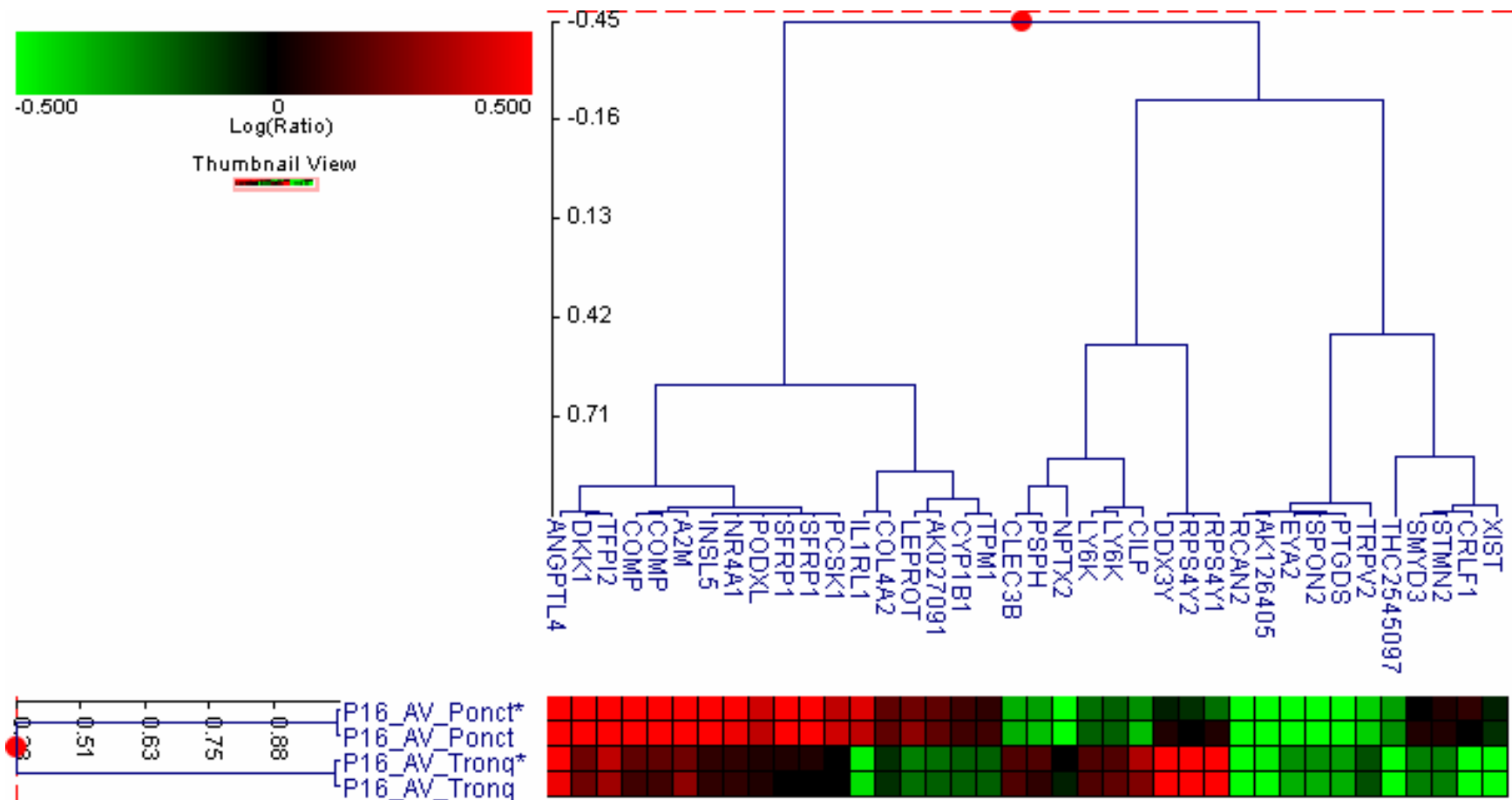

Supplement: Figure S1 — Cluster analysis of differentially expressed genes between the missense and the nonsense pools. Shown is the 2D cluster analysis of the ANOVA results of the microarray data (with p<10−10 as threshold). The log(ratio) for each slides of the dye-swap for the missense and nonsense pools are illustrated in red when the mRNAs are over-represented in the NBCCS pool compare to the control pool and conversely in green. The slides “missense and nonsense pools” marked with an asterisk (*) were incubated with Cy5 for the control target and Cy3 for the NBCCS target, and reciprocally for the slides without asterisk. Details on the genes and the ratios of intensity between NBCCS and control pools are included in Table S3. (0.01 MB PDF) [file pone.0004818.s001.pdf]
