# Supplementary material for: PTCH1 +/− Dermal Fibroblasts Isolated from Healthy Skin of Gorlin Syndrome Patients Exhibit Features of Carcinoma Associated Fibroblasts
Source: PLoS One. 2009 Mar 16;4(3):e4818. doi: 10.1371/journal.pone.0004818 (PMC2654107; doi:10.1371/journal.pone.0004818)
Supplement: Table S1 — Common NBCCS signature of the microarray results: 308 genes differentially expressed (p<10−5) in the two NBCCS pools compared to the control pool. The microarray assay was performed in dye-swap. The results of the dye-swaps were combined for the missense and the nonsense pools. For each gene or sequence, the fold change and its associated p-value are mentioned. Positive fold changes stand for an increased expression in NBCCS pool; negative fold changes stand for a decreased expression in NBCCS pool. (0.02 MB PDF) [file pone.0004818.s002.pdf]

Table S1

Primary Sequence Name	Accession Number	missense pool		nonsense pool	
		Fold Change	p-value	Fold Change	p-value
MMP3	NM_002422	19.7421	0	13.0139	0
CLIC6	NM_053277	18.5676	1.72E-35	7.711	3.54E-13
KCNJ8	NM_004982	12.2448	0	10.1035	5.14E-32
COL11A1	NM_080629	9.7967	9.06E-22	5.4611	4.28E-14
IL13RA2	NM_000640	8.9084	0	4.6296	2.00E-19
A2M	NM_000014	8.6147	0	1.7405	2.44E-06
MMP1	NM_002421	8.4122	0	7.8028	0
ANGPTL4	NM_139314	7.5098	0	3.1122	0
A_23_P170719		6.1947	0	2.6836	3.10E-24
TFPI2	AK129833	6.1688	4.05E-40	2.4071	1.13E-06
A_32_P75141		6.1635	0	4.9618	0
ALDH1A3	NM_000693	5.92	0	10.883	0
FLJ14834	NM_032849	5.8414	0	2.7641	6.60E-32
SMOC2	NM_022138	5.5702	6.90E-20	2.788	4.85E-06
TFPI2	NM_006528	5.533	0	2.0953	5.21E-21
PRL	NM_000948	5.4189	0	2.484	2.42E-15
AI476245	AI476245	5.1206	0	3.0481	3.41E-17
APBB1IP	NM_019043	4.6804	0	3.7532	2.47E-40
GHR	NM_000163	4.5814	9.60E-26	3.0934	4.03E-06
BX537788	BX537788	4.3842	9.63E-37	3.408	1.22E-15
DHRS3	NM_004753	4.3342	0	3.7553	3.85E-33
NR4A3	NM_173200	4.2339	0	2.1442	3.44E-11
MGST1	NM_145791	3.9293	0	2.5332	2.55E-24
A_24_P289854		3.7382	7.02E-36	3.5528	3.95E-18
CPM	NM_001874	3.715	0	2.1577	1.14E-25
NANOS1	NM_199461	3.609	3.75E-13	2.6956	5.74E-12
PPAP2A	NM_176895	3.5108	6.87E-38	2.23	1.83E-35
PCSK5	NM_006200	3.4846	0	2.1278	1.36E-09
GPC6	BX640888	3.4137	7.47E-36	2.405	7.60E-21
DKK1	NM_012242	3.3686	0	1.6358	3.03E-26
MASP1	NM_139125	3.2252	1.34E-21	3.421	3.33E-18
SPOCK1	NM_004598	3.1251	0	3.3341	0
CPM	NM_001874	3.1094	2.52E-19	2.0461	5.08E-08
FMOD	NM_002023	3.1081	0	1.8023	2.31E-16
KCTD15	NM_024076	3.0865	1.44E-21	3.0498	1.42E-19
SAA1	NM_000331	3.048	0	2.1112	1.25E-21
SNCA	NM_007308	3.0297	3.71E-30	2.5018	7.41E-09
MGC42157	BC030111	2.9857	0	2.759	1.59E-22
CA12	NM_001218	2.9836	0	2.1333	0
A_24_P752362		2.9768	8.03E-23	2.8958	2.09E-34
THC2140046	BG428517	2.927	0	2.0764	1.36E-24
STEAP1	NM_012449	2.9091	0	2.3808	7.46E-35
AKR1C1	NM_001353	2.8883	0	2.3474	1.23E-39
HSPA5BP1	NM_017870	2.8592	5.23E-18	2.2717	4.12E-15
STEAP1	NM_012449	2.8538	0	2.2024	2.10E-39
AKR1C1	NM_001353	2.8523	5.59E-37	2.4218	7.18E-21
AF264625	AF264625	2.8355	7.77E-28	2.8518	2.37E-25
PTGIS	NM_000961	2.8252	5.37E-22	2.7335	2.82E-23
STEAP2	NM_152999	2.8101	7.94E-37	2.2325	1.10E-09
CXCL12	NM_199168	2.8062	1.10E-34	2.5629	5.33E-25
A_24_P647682		2.699	3.75E-26	2.6545	1.51E-15
CDON	AK022986	2.6989	2.02E-19	2.2717	1.60E-08
LOC387763	BC052560	2.6507	3.76E-34	1.7848	1.72E-07
DPP3	NM_130443	2.6339	8.64E-18	2.6929	3.39E-17
SLC39A8	NM_022154	2.6101	3.18E-11	2.0025	9.21E-07
EFEMP1	NM_004105	2.5939	0	1.5247	1.01E-10
BC062473	BC062473	2.5805	4.85E-40	1.7842	1.50E-20
AW946823	AW946823	2.5737	1.71E-14	2.0896	7.27E-08
CTHRC1	NM_138455	2.5446	5.94E-20	1.6947	4.59E-07
ADFP	NM_001122	2.5132	4.54E-17	2.0442	4.12E-07
WISP2	NM_003881	2.5103	1.30E-23	2.1266	6.04E-18
A_24_P934306		2.4805	7.54E-15	2.6047	3.08E-11
SAA2	NM_030754	2.4569	0	1.9001	4.05E-15
COL12A1	NM_004370	2.3825	9.44E-37	1.6513	4.51E-10
TUBA1	NM_006000	2.3766	3.08E-27	2.0994	7.09E-10
CCRL1	NM_178445	2.3757	1.12E-18	2.4162	1.81E-26
AA417913	AA417913	2.37	3.52E-19	2.409	1.49E-29
AKR1C3	NM_003739	2.3671	8.04E-21	2.3569	7.55E-24
CMTM1	NM_181289	2.3129	3.71E-31	2.3448	1.11E-25
FGF7	NM_002009	2.3002	8.39E-30	1.5897	2.05E-18
FLJ30435	NM_174950	2.284	6.79E-22	1.4957	5.59E-07
EXOSC6	NM_058219	2.2792	0	2.3187	1.12E-44
ID2	NM_002166	2.2761	7.96E-41	1.6009	7.44E-17
SMAD3	NM_005902	2.2706	2.19E-25	1.8951	4.67E-18
DSCR1	NM_004414	2.2516	1.42E-10	2.4037	1.24E-11
UCHL1	NM_004181	2.2284	0	1.7156	1.55E-25

ADARB1	NM_015833	2.2018	6.96E-29	2.4494	6.38E-38
KIAA0644	AB014544	2.1862	1.50E-13	1.6568	9.87E-06
PLS3	NM_005032	2.1629	2.04E-41	1.3926	2.64E-09
LCE2A	NM_178428	2.1528	2.54E-35	2.0718	2.23E-17
BC035260	BC035260	2.1041	3.96E-12	2.4093	6.50E-09
QPCT	NM_012413	2.1039	8.56E-24	1.3834	3.07E-07
PITPNC1	NM_181671	2.0932	4.25E-08	2.0012	3.73E-06
ATP6V1B2	NM_001693	2.045	8.66E-17	2.0845	6.81E-15
MGP	NM_000900	2.0449	1.75E-14	1.4459	1.58E-07
QPCT	NM_012413	2.0333	1.26E-42	1.3246	5.90E-06
SAMD10	NM_080621	2.0178	6.03E-27	1.9777	9.01E-38
ANGPTL2	NM_012098	2.0155	2.91E-14	1.6564	6.67E-08
GALNTL2	NM_054110	2.0129	9.76E-41	1.5649	8.93E-09
AJ276555	AJ276555	2.0098	5.57E-16	2.046	4.57E-20
ABCC3	NM_003786	1.9821	1.14E-09	1.6254	6.40E-08
EMP2	NM_001424	1.9756	4.75E-20	1.7299	2.52E-22
SAT	NM_002970	1.9729	1.26E-21	1.7193	3.93E-10
SYNPO	AB028952	1.9568	4.59E-14	2.0189	3.50E-21
SFRP2	NM_003013	1.9555	4.84E-28	2.5881	3.79E-33
PGM1	NM_002633	1.9421	9.20E-14	1.9195	9.82E-17
ITGB5	NM_002213	1.9381	2.75E-17	1.5957	4.74E-07
AMID	NM_032797	1.902	7.82E-21	1.5964	8.03E-07
ID2	NM_002166	1.8965	7.60E-19	1.3594	2.40E-06
THBD	NM_000361	1.8919	1.34E-08	1.9982	1.26E-07
CUGBP2	NM_006561	1.8895	7.44E-24	1.8691	2.36E-28
MAB21L2	NM_006439	1.888	3.08E-13	1.8594	2.71E-20
SAT	NM_002970	1.8807	3.42E-28	1.7576	9.58E-19
HIG2	NM_013332	1.8775	1.12E-44	1.4082	1.38E-13
BNIP3	NM_004052	1.8772	2.21E-27	1.411	6.29E-09
GREM1	NM_013372	1.8693	4.03E-14	1.7254	4.64E-15
KIAA0664	NM_015229	1.857	1.91E-31	1.8454	3.04E-32
SOX4	NM_003107	1.8556	9.70E-14	1.6116	3.56E-08
THC2104265	AI358607	1.8521	1.36E-20	1.7994	1.51E-29
ANXA6	NM_001155	1.8518	2.33E-17	1.6998	1.91E-09
SFRP2	NM_003013	1.8437	1.14E-37	2.5863	0
BX537732	BX537732	1.8139	2.50E-13	1.6883	1.52E-10
AA516273	AA516273	1.8088	1.28E-06	1.7599	3.24E-07
BC031278	BC031278	1.7963	4.22E-22	1.7613	1.68E-26
COL12A1	NM_004370	1.783	4.86E-15	1.6408	1.47E-10
DPYSL4	NM_006426	1.7711	6.96E-10	1.61	1.01E-07
CD44	NM_000610	1.7313	1.91E-12	1.5131	5.27E-06
LEPROT	NM_017526	1.727	7.73E-16	-1.5699	5.37E-11
C1orf42	NM_019060	1.7216	1.16E-12	1.6265	1.08E-09
C1GALT1C1	NM_152692	1.7104	7.56E-18	1.4669	1.64E-09
LMOD1	NM_012134	1.709	8.39E-10	1.7694	8.53E-10
EMP2	AK096403	1.7085	8.01E-14	1.6664	1.39E-16
E2IG5	NM_014367	1.7059	6.19E-18	1.3819	1.00E-07
THC2100006		1.6642	6.75E-12	1.6337	4.60E-09
C20orf19	NM_018474	1.6635	1.96E-12	1.5786	1.30E-10
EMX2OS	AY117034	1.6578	5.59E-11	1.8749	5.76E-16
BASP1	NM_006317	1.6281	4.33E-10	1.6043	4.29E-09
NT5E	NM_002526	1.6194	1.80E-08	1.7122	1.21E-10
CDH13	NM_001257	1.611	7.47E-07	2.1522	2.60E-15
SOD2	NM_000636	1.6025	1.15E-19	1.7405	1.10E-20
SMPDL3A	NM_006714	1.5995	1.41E-15	1.396	6.76E-07
AOX1	NM_001159	1.5893	1.80E-18	1.5901	3.26E-19
CARHSP1	NM_014316	1.5807	7.01E-06	1.8301	3.00E-13
BTG1	NM_001731	1.5781	7.71E-08	1.6958	4.54E-07
SVEP1	AK075235	1.5754	9.36E-13	1.5834	9.27E-17
TNXB	NM_019105	1.5661	3.94E-07	2.8929	8.75E-36
CYR61	NM_001554	1.5646	9.26E-24	1.2641	2.19E-08
GPT	NM_005309	1.5459	3.93E-25	1.5546	3.00E-16
HSPB2	NM_001541	1.5429	1.13E-08	1.43	3.40E-07
KLF6	NM_001300	1.5373	4.44E-23	1.3389	9.78E-10
EMX2	NM_004098	1.5242	5.28E-06	1.7484	1.71E-08
NDN	NM_002487	1.5226	2.98E-08	1.5537	1.53E-09
KRTAP5-8	NM_021046	1.522	3.46E-13	1.4603	6.31E-09
THC2230875		1.5103	2.79E-13	1.4767	8.09E-13
X68990	X68990	1.5084	6.73E-16	1.4834	1.07E-16
RECK	NM_021111	1.508	1.12E-06	1.455	2.05E-07
GLRX	NM_002064	1.5039	8.74E-20	1.3365	1.71E-08
DIRC1	NM_052952	1.5025	6.06E-20	1.421	4.71E-09
PDPN	NM_006474	1.5016	6.68E-07	1.9428	1.23E-26
PAM	NM_000919	1.4992	1.03E-11	1.497	1.55E-09
FKBP10	NM_021939	1.4919	3.32E-11	1.4244	6.35E-11
EDARADD	NM_145861	1.4836	7.08E-11	1.339	1.16E-10
TNFAIP2	NM_006291	1.4815	2.03E-18	1.4017	3.86E-08
ENST00000296496	CR597270	1.472	2.94E-08	1.2854	7.44E-06
CD99	NM_002414	1.4622	3.18E-09	1.3388	7.41E-10

MT	NM_014507	1.4584	5.18E-20	1.3918	6.95E-11
PRSS36	NM_173502	1.4489	9.07E-11	1.4285	2.60E-14
IL27	NM_145659	1.4436	1.07E-13	1.3817	2.70E-11
AF155662	AF155662	1.4373	1.26E-07	1.5008	4.04E-10
PAM	NM_000919	1.4362	3.12E-14	1.4177	5.03E-10
REV3L	NM_002912	1.4361	1.75E-06	1.5779	6.18E-07
ZFP36L2	NM_006887	1.4351	3.82E-16	1.4675	2.11E-17
SGCE	NM_003919	1.4298	9.05E-08	1.4387	8.35E-06
RPLP0	BF570115	1.4295	1.79E-09	1.593	1.02E-09
KIAA1199	NM_018689	1.4213	8.36E-09	1.6891	4.35E-12
LOC441245	AK090474	1.4213	3.06E-10	1.3605	3.93E-10
COPS2	NM_004236	1.4181	2.95E-06	1.4172	1.64E-07
EMILIN2	NM_032048	1.4158	1.68E-13	1.4087	1.12E-13
TNC	NM_002160	1.4026	1.67E-10	1.3453	2.12E-06
ZBED3	NM_032367	1.4	2.62E-07	1.4781	6.17E-06
ARL6IP5	NM_006407	1.3919	8.81E-08	1.4065	1.34E-09
DLST	NM_001933	1.377	9.10E-13	1.4567	7.80E-15
THC2088849		1.3662	5.54E-11	1.4553	5.50E-12
FLJ11125	AK001987	1.3603	6.21E-08	1.3274	2.94E-11
PLA2G4A	NM_024420	1.3564	2.86E-07	2.2496	0
KIAA1199	NM_018689	1.3422	1.19E-07	1.6131	1.63E-15
BM921275	BM921275	1.3403	3.58E-06	1.3433	2.70E-07
A_24_P384210		1.3293	8.66E-10	1.2797	1.77E-06
CYP1B1	NM_000104	1.3155	1.74E-10	-1.4357	6.11E-16
COLEC12	NM_030781	1.275	9.63E-07	1.8187	7.71E-17
CTSB	AK097384	1.2593	3.13E-07	1.4216	6.47E-08
TPCN1	AK000619	1.2438	1.53E-06	1.6269	8.66E-15
RAB31	NM_006868	-1.2302	8.20E-06	-1.4304	1.88E-09
COL1A1	NM_000088	-1.2561	8.93E-07	-1.4463	6.55E-13
FBLN2	NM_001998	-1.259	9.61E-08	-1.2602	4.53E-06
HTRA1	NM_002775	-1.2794	7.07E-06	-1.3294	1.33E-09
C1orf188	NM_173795	-1.2827	8.52E-09	-1.2649	2.08E-07
PYCR1	NM_153824	-1.2966	6.64E-06	-1.3304	3.66E-08
FBN1	NM_000138	-1.3008	5.64E-09	-1.3062	1.59E-06
C6orf48	NM_016947	-1.317	9.10E-08	-1.2647	2.96E-07
SLC20A1	NM_005415	-1.3303	3.16E-11	-1.2623	1.80E-06
PRNP	NM_000311	-1.3309	5.69E-12	-1.3382	1.51E-10
SERP1	NM_014445	-1.3356	5.69E-08	-1.3277	3.49E-06
IL15	NM_172174	-1.3507	2.18E-10	-1.3858	1.92E-06
COL3A1	NM_000090	-1.3649	4.75E-11	-1.3879	1.33E-07
LY6K	NM_017527	-1.3724	1.36E-12	1.4197	7.08E-10
LOC388610	BC069216	-1.3732	2.17E-07	-1.3316	1.49E-10
ADH1A	NM_000667	-1.3742	6.22E-06	-1.3893	1.96E-08
RAB31	NM_006868	-1.3985	1.65E-15	-1.3186	1.12E-07
LY6K	NM_017527	-1.4	5.68E-08	1.3563	8.97E-12
CHST2	NM_004267	-1.4066	4.42E-07	-1.6757	4.72E-08
THC2051764		-1.4189	3.56E-07	-1.4977	6.31E-09
ADAM12	NM_003474	-1.4238	2.64E-10	-1.5128	4.89E-06
C9orf150	NM_203403	-1.4285	7.33E-09	-1.6064	1.34E-07
WIPI1	NM_017983	-1.4296	2.05E-09	-1.3844	3.39E-07
COL3A1	NM_000090	-1.4326	6.65E-09	-1.5389	5.69E-12
THC2042343		-1.437	5.90E-12	-1.2269	4.42E-06
NDUFA12	NM_018838	-1.4427	1.91E-10	-1.3934	2.95E-08
GRB10	NM_001001555	-1.4508	3.02E-16	-1.3402	1.81E-06
CXCL2	NM_002089	-1.4518	3.83E-08	-2.0825	1.99E-08
CR617391	CR617391	-1.4559	4.50E-09	-1.8749	8.05E-27
COL5A1	NM_000093	-1.4651	1.11E-13	-1.7266	2.36E-26
FDXR	NM_004110	-1.4703	1.18E-06	-1.3251	4.18E-09
LAYN	NM_178834	-1.4764	4.06E-07	-1.4906	3.51E-13
COL1A2	NM_000089	-1.4806	9.14E-12	-1.3149	8.51E-08
BAX	NM_138764	-1.4923	1.28E-07	-1.3944	8.05E-07
LOXL3	NM_032603	-1.4986	1.39E-07	-2.0143	9.42E-27
PDIA3	NM_005313	-1.5006	4.06E-08	-1.5565	1.59E-07
MALAT1	BX538238	-1.5079	9.29E-15	-1.45	1.11E-12
THC2054763		-1.5113	2.97E-09	-1.8521	2.73E-06
GLT8D2	NM_031302	-1.5234	1.62E-09	-1.5828	2.18E-06
LRAP	NM_022350	-1.5236	1.47E-07	-2.1474	2.18E-09
C14orf147	NM_138288	-1.5243	1.08E-09	-1.4347	4.55E-10
PTPRK	NM_002844	-1.5308	8.24E-12	-1.3486	3.46E-06
ACOT2	NM_006821	-1.5324	2.26E-11	-1.4288	1.39E-06
LAMA2	NM_000426	-1.5328	2.11E-09	-1.4533	7.55E-09
ASNS	NM_001673	-1.5371	3.94E-20	-1.4911	1.58E-15
LOC389652	XM_372040	-1.5413	1.23E-11	-1.5574	1.76E-15
CDC42EP3	NM_006449	-1.5728	1.15E-07	-1.5648	1.42E-10
INHBB	NM_002193	-1.5886	8.37E-26	-1.5278	1.13E-16
AK055915	AK055915	-1.5952	4.73E-14	-1.5486	1.08E-06
MARVELD1	NM_031484	-1.5971	8.69E-11	-1.6071	1.12E-09
FST	NM_013409	-1.5995	1.22E-17	-1.5192	1.61E-09
TncRNA	AK027191	-1.6019	7.49E-11	-1.4575	1.98E-13

RUTBC3	NM_015705	-1.6092	3.51E-06	-1.626	4.81E-07
PHLDA1	NM_007350	-1.6221	6.42E-24	-1.5427	1.52E-14
PDGFRB	NM_002609	-1.6261	4.06E-20	-1.4109	1.15E-12
SCARB1	NM_005505	-1.6479	3.66E-21	-1.6919	8.39E-15
LOC442293	XM_498178	-1.6856	2.53E-10	-1.6404	1.30E-07
LRIG3	NM_153377	-1.6875	1.65E-17	-1.4743	4.33E-10
CLEC2B	NM_005127	-1.6982	8.23E-26	-1.6084	8.22E-16
ITGBL1	NM_004791	-1.716	9.16E-19	-1.9716	1.56E-27
CXCL1	NM_001511	-1.7183	3.77E-07	-2.1977	6.06E-12
BF213738	BF213738	-1.7398	4.65E-19	-1.6952	4.92E-32
BC066344	BC066344	-1.7401	2.21E-18	-1.7629	3.65E-10
FPR1	NM_002029	-1.741	9.19E-20	-1.6412	2.19E-09
PSAT1	NM_058179	-1.7732	1.28E-18	-1.825	4.65E-21
A_23_P255111		-1.7949	4.89E-14	-5.2354	0
IGFBP7	NM_001553	-1.891	2.89E-06	-6.0029	0
CILP	NM_003613	-1.9239	2.27E-09	1.9266	3.45E-08
CHST7	NM_019886	-1.9272	2.91E-26	-2.4096	4.61E-28
LFNG	BC014851	-1.9288	4.82E-12	-1.6955	1.12E-06
MUC20	NM_152673	-1.936	1.15E-06	-2.4596	1.41E-21
APOE	NM_000041	-1.9402	1.11E-42	-1.3029	5.30E-08
MLPH	NM_024101	-1.9409	1.83E-16	-1.7616	1.33E-09
AK026784	AK026784	-1.9424	1.36E-10	-2.0477	5.27E-10
AK075484	AK075484	-1.9695	9.44E-21	-2.0015	6.05E-19
AL359052	AL359052	-2.0027	7.30E-16	-2.0856	1.69E-13
SERPINF1	NM_002615	-2.0094	9.40E-09	-1.6694	3.12E-06
CLEC3B	NM_003278	-2.016	0	1.3543	2.05E-13
THC2173240		-2.0246	2.89E-09	-1.6599	2.17E-10
MRPS6	NM_032476	-2.0451	7.21E-12	-1.6673	4.36E-11
CHN1	NM_001822	-2.0453	1.34E-16	-2.1094	4.55E-20
LOC375295	BC013438	-2.0584	7.35E-12	-2.4154	2.93E-16
CH25H	NM_003956	-2.062	0	-1.6382	4.24E-14
THC2208133	AI620901	-2.0711	2.25E-11	-1.9838	9.53E-08
NET1	NM_005863	-2.1241	3.03E-31	-1.4669	1.45E-08
LUM	NM_002345	-2.1416	0	-2.1803	3.21E-31
SPSB2	NM_032641	-2.1485	1.91E-12	-2.1705	1.94E-14
IGFBP2	NM_000597	-2.1809	7.33E-30	-4.2923	0
CPT1C	NM_152359	-2.1895	5.27E-42	-1.4124	8.16E-06
DKFZp434L142	NM_016613	-2.2053	0	-1.8556	1.41E-22
BC037430	BC037430	-2.2074	4.78E-30	-1.9038	7.93E-18
pp9099	NM_025201	-2.2115	1.39E-20	-1.5228	1.59E-07
NOPE	NM_020962	-2.2406	6.31E-09	-2.1367	1.82E-07
RSPO3	NM_032784	-2.2446	4.25E-10	-2.7686	1.58E-14
TRPV2	NM_016113	-2.258	0	-1.2706	9.04E-06
MYLK	NM_053025	-2.2814	1.44E-16	-2.2724	1.14E-17
CSPG4	NM_001897	-2.3191	2.53E-16	-1.9602	4.89E-07
SFRS5	NM_006925	-2.334	1.06E-37	-2.1145	1.68E-42
MYLK	NM_053025	-2.3385	3.19E-12	-2.1422	2.05E-09
CSRP2	NM_001321	-2.3663	1.94E-30	-1.6951	2.09E-13
LOC375295	BC013438	-2.4275	3.41E-20	-2.7879	1.25E-14
EVC	NM_014556	-2.5052	1.34E-12	-1.6697	7.79E-08
MRPS6	NM_032476	-2.5203	1.31E-25	-2.004	6.07E-23
FLJ10157	AK001019	-2.5374	3.10E-10	-3.7488	1.34E-18
ZNF179	NM_007148	-2.5971	1.42E-25	-1.7311	1.70E-06
WNT5A	NM_003392	-2.9356	0	-2.714	0
CSEN	NM_013434	-3.072	0	-2.0124	1.27E-29
TBX5	NM_000192	-3.1129	3.38E-12	-3.536	1.15E-07
ADH4	NM_000670	-3.23	8.12E-12	-3.827	4.91E-12
BST2	NM_004335	-3.2752	0	-3.0303	1.29E-15
DKK3	NM_013253	-3.3895	6.82E-26	-1.9717	3.39E-10
DKK3	NM_015881	-3.5023	0	-1.9682	1.97E-11
COL23A1	NM_173465	-3.6652	7.01E-45	-2.1148	4.82E-14
AQP1	NM_000385	-3.6666	0	-2.5611	2.90E-36
PTGDS	NM_000954	-3.9356	0	-1.9318	9.78E-25
IFI27	NM_005532	-4.1376	7.34E-18	-4.3466	4.27E-15
IFI27	NM_005532	-4.1996	0	-4.7571	0
ECHDC3	NM_024693	-4.6909	1.88E-23	-5.9668	1.37E-24
EYA2	NM_172113	-4.8738	0	-1.901	7.22E-10
SPON2	NM_012445	-4.953	0	-1.8769	5.91E-13
TBX1	NM_080647	-5.1205	0	-4.7772	0
AK126405	AK126405	-5.9166	0	-2.7683	0
CPXM2	NM_198148	-5.9272	2.66E-44	-2.223	7.27E-09
TNFRSF19	NM_148957	-6.1012	6.96E-32	-3.8355	9.09E-15
THC2105523		-7.1402	0	-5.2897	0
DSCR1L1	NM_005822	-9.6226	0	-3.713	0
