# Supplementary material for: PTCH1 +/− Dermal Fibroblasts Isolated from Healthy Skin of Gorlin Syndrome Patients Exhibit Features of Carcinoma Associated Fibroblasts
Source: PLoS One. 2009 Mar 16;4(3):e4818. doi: 10.1371/journal.pone.0004818 (PMC2654107; doi:10.1371/journal.pone.0004818)
Supplement: Table S2 — Anti-correlated genes between the missense and the nonsense pools. List of the genes up-regulated in one NBCCS pool and down regulated in the other among the genes with differential expression in NBCCS pools compare to the control pool (p<10−5). For each gene, the fold change and its associated p-value are mentioned. Positive fold changes stand for an increased expression in NBCCS pool; negative fold changes stand for a decreased expression in NBCCS pool. (0.01 MB PDF) [file pone.0004818.s003.pdf]

Table S2

Primary Sequence Name	Accession Number	missense pool		nonsense pool	
		Fold Change	p-value	Fold Change	p-value
CILP	NM_003613	-1.9239	2.27E-09	1.9266	3.45E-08
LY6K	NM_017527	-1.3724	1.36E-12	1.4197	7.08E-10
LY6K	NM_017527	-1.4	5.68E-08	1.3563	8.97E-12
CLEC3B	NM_003278	-2.016	0	1.3543	2.05E-13
CYP1B1	NM_000104	1.3155	1.74E-10	-1.4357	6.11E-16
LEPROT	NM_017526	1.727	7.73E-16	-1.5699	5.37E-11
