# Supplementary material for: PTCH1 +/− Dermal Fibroblasts Isolated from Healthy Skin of Gorlin Syndrome Patients Exhibit Features of Carcinoma Associated Fibroblasts
Source: PLoS One. 2009 Mar 16;4(3):e4818. doi: 10.1371/journal.pone.0004818 (PMC2654107; doi:10.1371/journal.pone.0004818)
Supplement: Table S3 — 38 differentially expressed genes between the missense and the nonsense pools found by analysis of variance of the microarray results. For each slide of the dye-swaps, the fold change between NBCCS and control pools are indicated for the 38 genes differentially expressed between the two NBCCS pools. Positive fold changes stand for an increased expression in NBCCS pools; negative fold changes stand for a decreased expression in NBCCS pools. The slides “missense and nonsense pools” marked with an asterisk (*) were incubated with Cy5 for the control target and Cy3 for the NBCCS target, and reciprocally for the slides without asterisk. (0.01 MB PDF) [file pone.0004818.s004.pdf]

Table S3

Primary Sequence Name	Sequence Description	Accession Number	Missense pool*	Missense pool	Nonsense pool*	Nonsense pool
ANGPTL4	angiopoietin-like 4	NM_139314	7.462	7.558	3.268	2.977
DKK1	dickkopf homolog 1 (Xenopus laevis)	NM_012242	3.506	3.243	1.644	1.628
TFPI2	tissue factor pathway inhibitor 2	NM_006528	5.77	5.317	2.241	1.969
COMP	cartilage oligomeric matrix protein	NM_000095	5.757	5.239	1.541	1.269
COMP	cartilage oligomeric matrix protein	NM_000095	6.001	4.613	1.532	1.28
A2M	alpha-2-macroglobulin	NM_000014	8.545	8.687	1.603	1.834
INSL5	insulin-like 5	NM_005478	5.662	5.424	1.178	1.23
NR4A1	nuclear receptor subfamily 4, group A, member 1	NM_002135	3.232	2.969	1.165	1.172
PODXL	podocalyxin-like	NM_001018111	2.496	2.28	1.092	1.082
SFRP1	secreted frizzled-related protein 1	NM_003012	4.41	3.666	1.128	1.018
SFRP1	secreted frizzled-related protein 1	NM_003012	4.041	3.6	1.132	1.058
PCSK1	proprotein convertase subtilisin/kexin type 1	NM_000439	2.306	2.446	1.06	1.023
IL1RL1	interleukin 1 receptor-like 1	NM_016232	2.673	2.391	-2.767	-2.568
COL4A2	collagen, type IV, alpha 2	NM_001846	1.472	1.463	-1.102	-1.101
LEPROT	leptin receptor overlapping transcript	NM_017526	1.648	1.814	-1.649	-1.501
AK027091	Homo sapiens cDNA: FLJ23438 fis, clone HRC13275. [AK027091]	AK027091	1.496	1.518	-1.522	-1.406
CYP1B1	cytochrome P450, family 1, subfamily B, polypeptide 1	NM_000104	1.32	1.311	-1.431	-1.44
TPM1	tropomyosin 1 (alpha)	NM_001018004	1.218	1.265	-1.369	-1.37
CLEC3B	C-type lectin domain family 3, member B	NM_003278	-2.021	-2.011	1.353	1.355
PSPH	phosphoserine phosphatase	NM_004577	-1.909	-2.118	1.378	1.224
NPTX2	neuronal pentraxin II	NM_002523	-9.093	-8.933	1.045	-1.021
LY6K	lymphocyte antigen 6 complex, locus K	NM_017527	-1.459	-1.345	1.347	1.366
LY6K	lymphocyte antigen 6 complex, locus K	NM_017527	-1.378	-1.366	1.379	1.461
CILP	cartilage intermediate layer protein, nucleotide pyrophosphohydrolase	NM_003613	-1.768	-2.119	2.22	1.735
DDX3Y	DEAD (Asp-Glu-Ala-Asp) box polypeptide 3, Y-linked	NM_004660	-1.001	1.085	7.796	6.871
RPS4Y2	ribosomal protein S4, Y-linked 2	NM_001039567	-1.141	1.053	48.763	48.704
RPS4Y1	ribosomal protein S4, Y-linked 1	NM_001008	-1.477	1.15	50.953	76.205
RCAN2	regulator of calcineurin 2	NM_005822	-9.243	-10.051	-3.604	-3.816
AK126405	Homo sapiens cDNA FLJ44441 fis, clone UTERU2020242. [AK126405]	AK126405	-6.002	-5.833	-2.67	-2.868
EYA2	eyes absent homolog 2 (Drosophila)	NM_172113	-4.856	-4.892	-1.742	-2.033
SPON2	spondin 2, extracellular matrix protein	NM_012445	-4.633	-5.33	-1.729	-2.05
PTGDS	prostaglandin D2 synthase 21kDa (brain)	NM_000954	-3.978	-3.895	-1.858	-2.011
TRPV2	transient receptor potential cation channel, subfamily V, member 2	NM_016113	-2.251	-2.265	-1.248	-1.292
THC2545097	IBP7_HUMAN (Q16270) Insulin-like growth factor-binding protein 7 precursor (IGFBP-7) (IBP-7) (IGF-binding protein		-1.92	-1.683	-5.476	-5.029
SMYD3	SET and MYND domain containing 3	NM_022743	1.073	1.086	-1.575	-1.553
STMN2	stathmin-like 2	S82024	1.142	1.08	-1.609	-1.621
CRLF1	cytokine receptor-like factor 1	NM_004750	1.188	1.028	-4.345	-4.678
XIST	Homo sapiens X (inactive)-specific transcript (XIST) on chromosome X.	NR_001564	-1.003	-1.083	-2.919	-3.185
