# Supplementary material for: PTCH1 +/− Dermal Fibroblasts Isolated from Healthy Skin of Gorlin Syndrome Patients Exhibit Features of Carcinoma Associated Fibroblasts
Source: PLoS One. 2009 Mar 16;4(3):e4818. doi: 10.1371/journal.pone.0004818 (PMC2654107; doi:10.1371/journal.pone.0004818)
Supplement: Table S4 — Primers used for quantitative real-time PCR. List of the TaqMan® Gene Expression Assays primers used for Q-PCR (Applied Biosystems, Foster City, USA, CA). (0.01 MB PDF) [file pone.0004818.s005.pdf]

Table S4

gene name	TaqMan® Gene Expression Assay Reference
MMP1	Hs00233958_m1
MMP3	Hs00233962_m1
COL3A1	Hs00943809_m1
COL7A1	Hs00164310_m1
COL11A1	Hs00266273_m1
LAMA2	Hs00166308_m1
FGF7	Hs00940253_m1
GREM1	Hs00171951_m1
MGP	Hs00179899_m1
CXCL12	Hs00171022_m1
ANGPTL2	Hs00765775_m1
ANGPTL4	Hs00211522_m1
WNT5A	Hs00180103_m1
SFRP2	Hs00293258_m1
DKK3	Hs00247426_m1
ID2	Hs00747379_m1
WISP2	Hs00180242_m1
TNC	Hs01115664_m1
TBP	Hs99999910_m1
GAPDH	Hs99999905_m1
PPIA	Hs99999904_m1
RPLO1	Hs99999902_m1
B2M	Hs99999907_m1
